# Supplementary figures and images for: Establishment of quantitative RNAi-based forward genetics in Entamoeba histolytica and identification of genes required for growth
Source: PLoS Pathog. 2021 Nov 29;17(11):e1010088. doi: 10.1371/journal.ppat.1010088 (PMC8716031; doi:10.1371/journal.ppat.1010088)

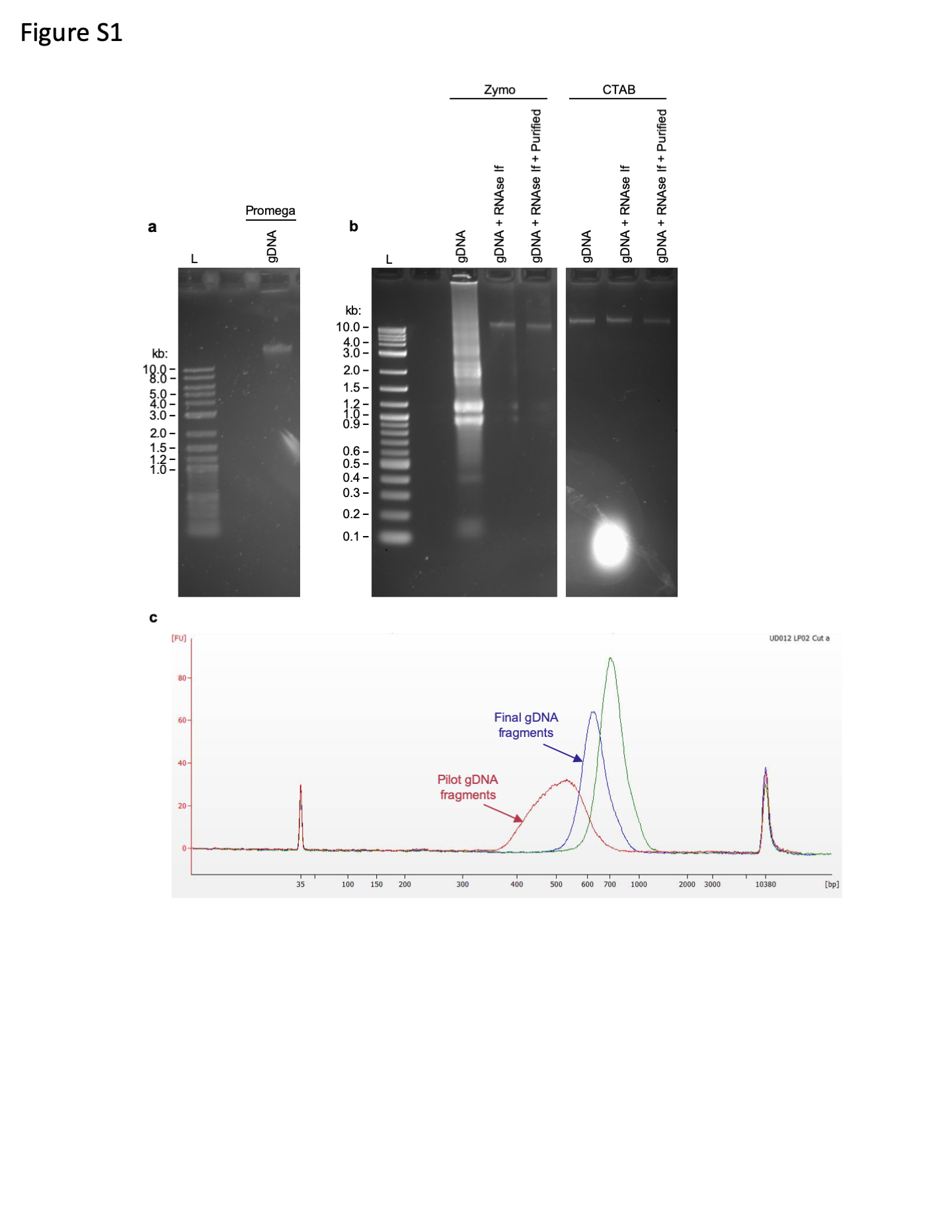

Supplement: S1 Fig — Gel electrophoresis analysis of gDNA extracted using the Wizard gDNA extraction kit (Promega), Quick-DNA miniprep kit (Zymo), or CTAB extraction. a, gDNA isolated using the Wizard kit had no apparent RNA contamination, and the gDNA appeared to be of high quality. b, gDNA isolated using the Quick-DNA kit or CTAB was contaminated with RNA. RNA contamination was removed by treatment with RNAseIF and subsequent purification. c, Bioanalyzer traces of pilot and final gDNA samples, after fragmentation and size selection. (TIFF) [file ppat.1010088.s001.tiff]

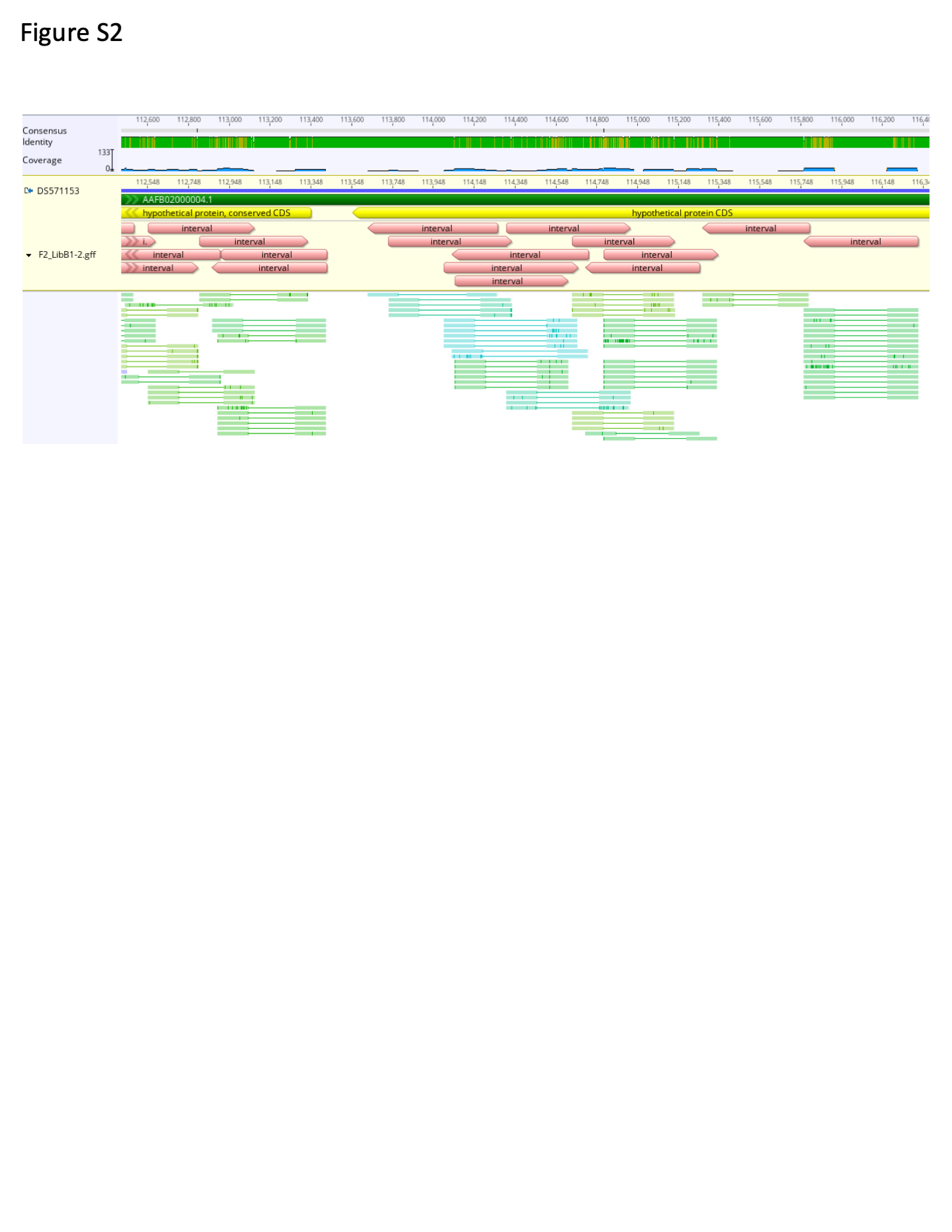

Supplement: S2 Fig — Example view of mapped genomic fragments (“intervals”) represented in the final plasmid library. For comparison, the mapped paired end reads that give rise to each fragment are shown below each fragment. Shown are results from final plasmid batch two, sequencing replicate one, mapped to contig DS571153. (TIFF) [file ppat.1010088.s002.tiff]

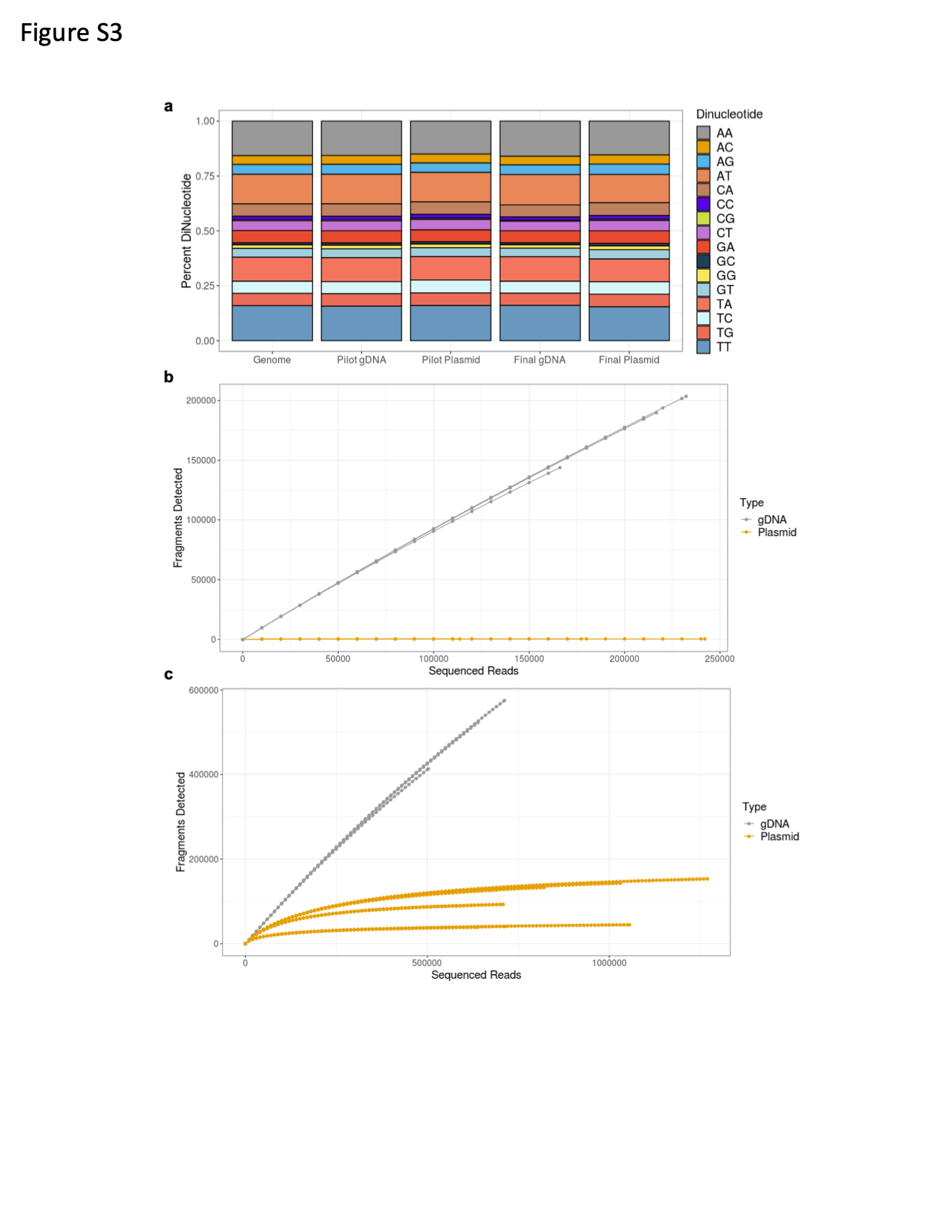

Supplement: S3 Fig — a, Dinucleotide frequency analysis of the HM1:IMSS reference genome, the pilot and final gDNA fragments, and the pilot and final plasmid libraries. Stacked bar plots show the percentage of each dinucleotide. The dinucleotide composition of the gDNA fragments and the final plasmid library is the same as the genome. b–c, Rarefaction curves for pilot (panel b) and final (panel c) samples. gDNA fragment samples are shown in grey and plasmid samples are shown in orange. The number of fragments detected with increasing sequence reads shows if sequencing was performed to saturation. Three sequencing replicates are shown for pilot gDNA samples and three sequencing replicates are shown for pilot plasmid samples. Three sequencing replicates are shown for final gDNA samples and three sequencing replicates are shown for each of four batches of final plasmid samples. (TIFF) [file ppat.1010088.s003.tiff]

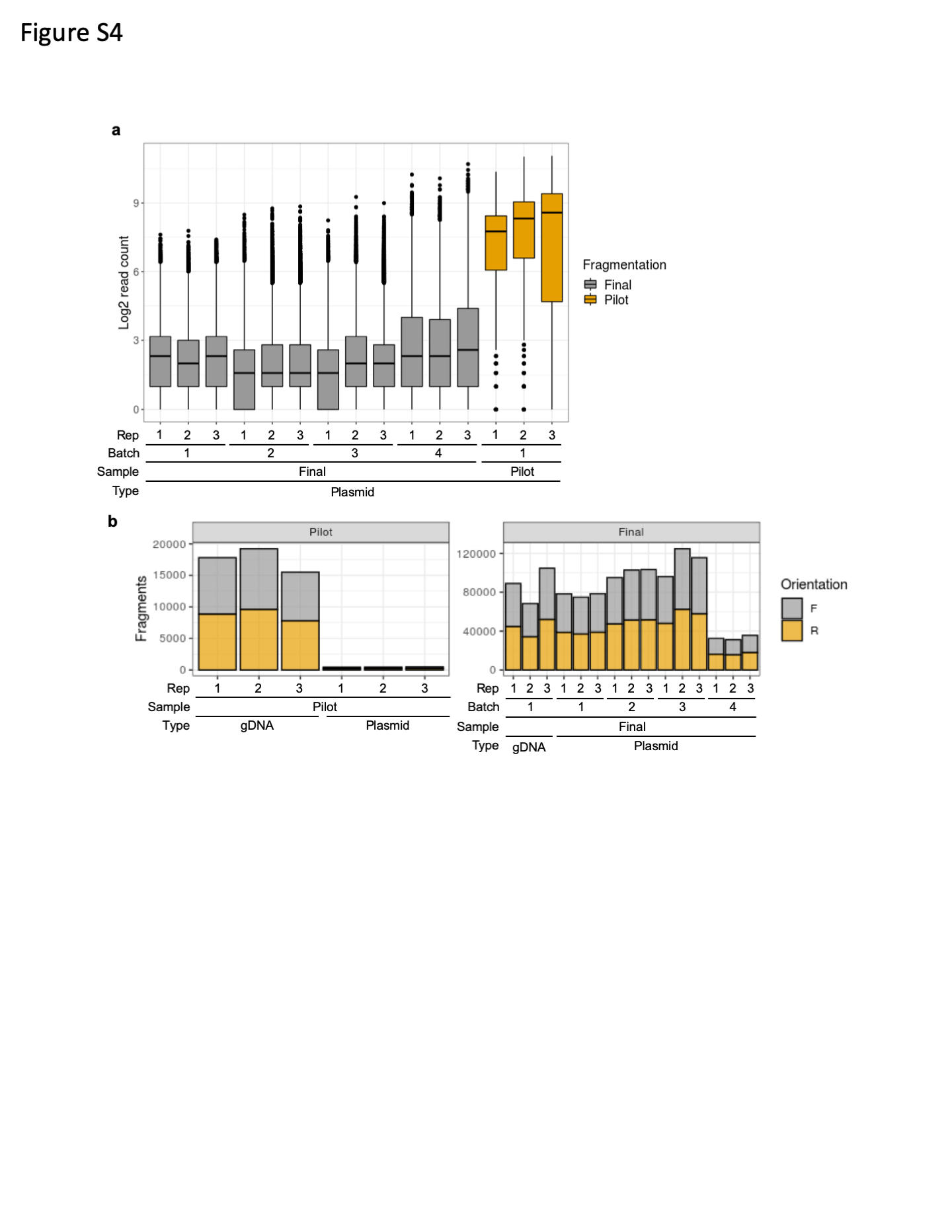

Supplement: S4 Fig — “Batches” of the final plasmid library were cloned on different days, and represent biological replicates. “Reps” correspond to independently prepared sequencing preparations, and represent technical replicates. a, Box plot of read counts per fragment in plasmid samples, with pilot plasmids in orange and final plasmids in grey. Log2 read counts are shown; outliers (outside of 2nd and 98th percentile) are plotted as points, and the box represents the 25th, and 75th percentile with median indicated as a bold line. Most fragments were represented by multiple sequenced reads. b, Stacked bar plots of the total number of fragments detected per sample shows that replicates are similar to each other in the number of fragments identified and that roughly equal numbers of forward and reverse fragments were produced consistently. Forward fragments are in the same orientation as genes that are in the forward orientation in the reference genome. Reverse fragments are in the same orientation as genes that are in the reverse orientation in the reference genome. (TIFF) [file ppat.1010088.s004.tiff]

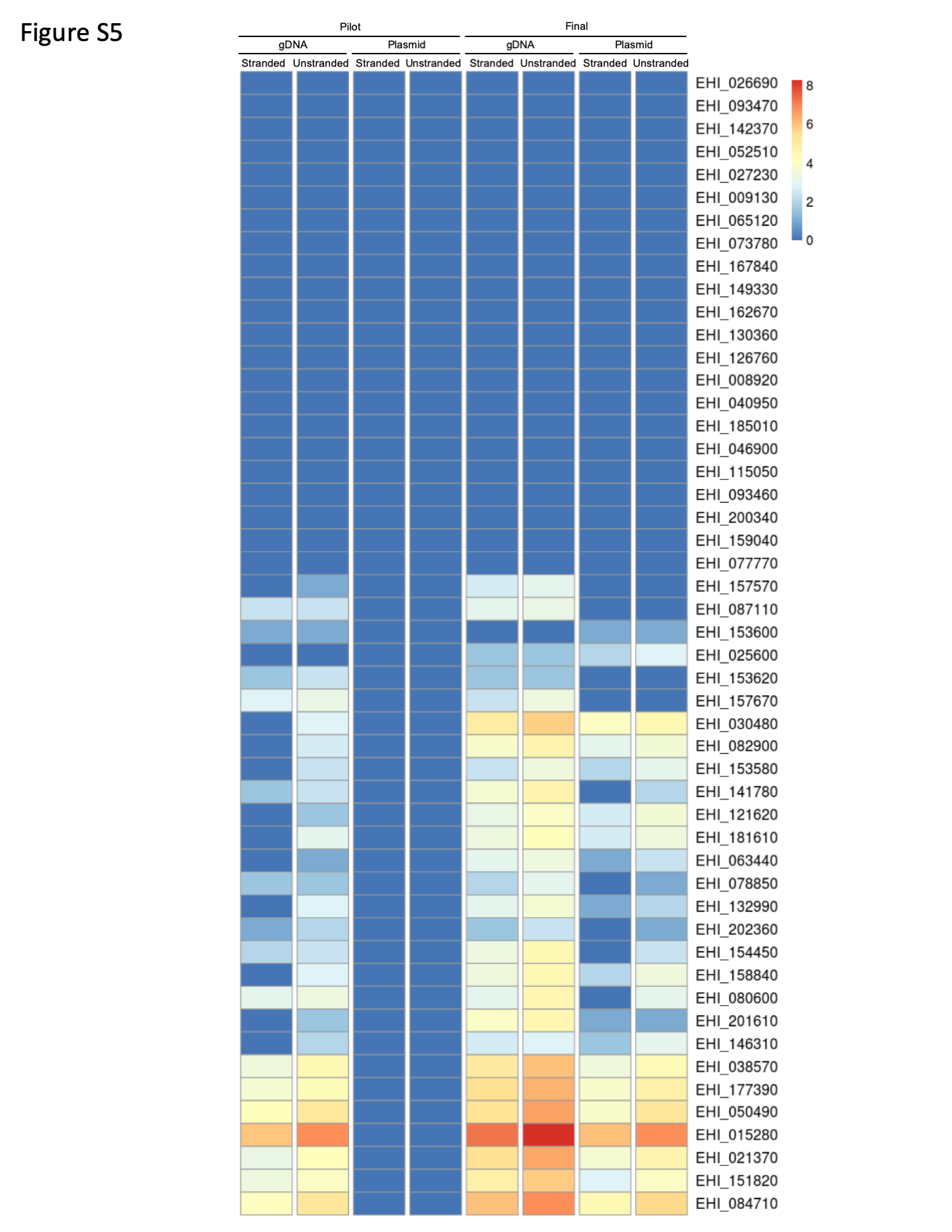

Supplement: S5 Fig — Heat map showing the number of unique fragments per gene, for the 50 least covered genes in the pilot and final gDNA fragments, and the pilot and final plasmid libraries. The color intensity indicates Log2 fragment coverage, with dark blue indicating no coverage. Stranded fragments are in the same orientation as genes. Unstranded fragments are the total number of fragments, both in the same orientation as genes and in the opposite orientation as genes. (TIFF) [file ppat.1010088.s005.tiff]

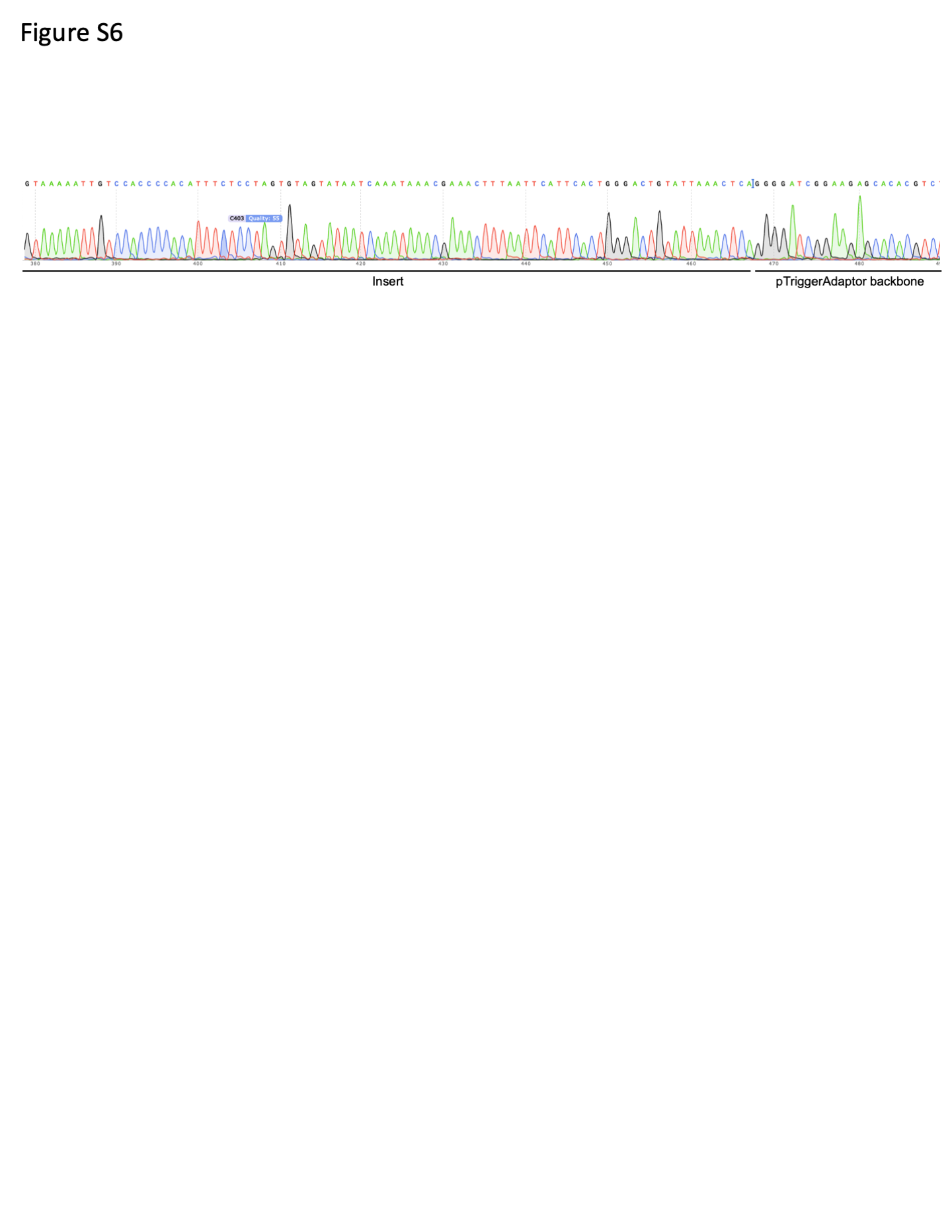

Supplement: S6 Fig — SG1 gDNA was extracted using the Quick-DNA miniprep kit and PCR amplified using the CS5’ and CS3’ primers that sit in the pTriggerAdaptor backbone. Sanger sequencing was performed using the RNAi Sequencing Library F primer. Adaptor 2 starts at the “GGGG” series of nucleotides, and upstream of this is the SG1 (EHI_194310) sequence. Noncompeting peaks in the chromatogram suggest that there is one unique genome fragment present in this mutant. (TIFF) [file ppat.1010088.s006.tiff]

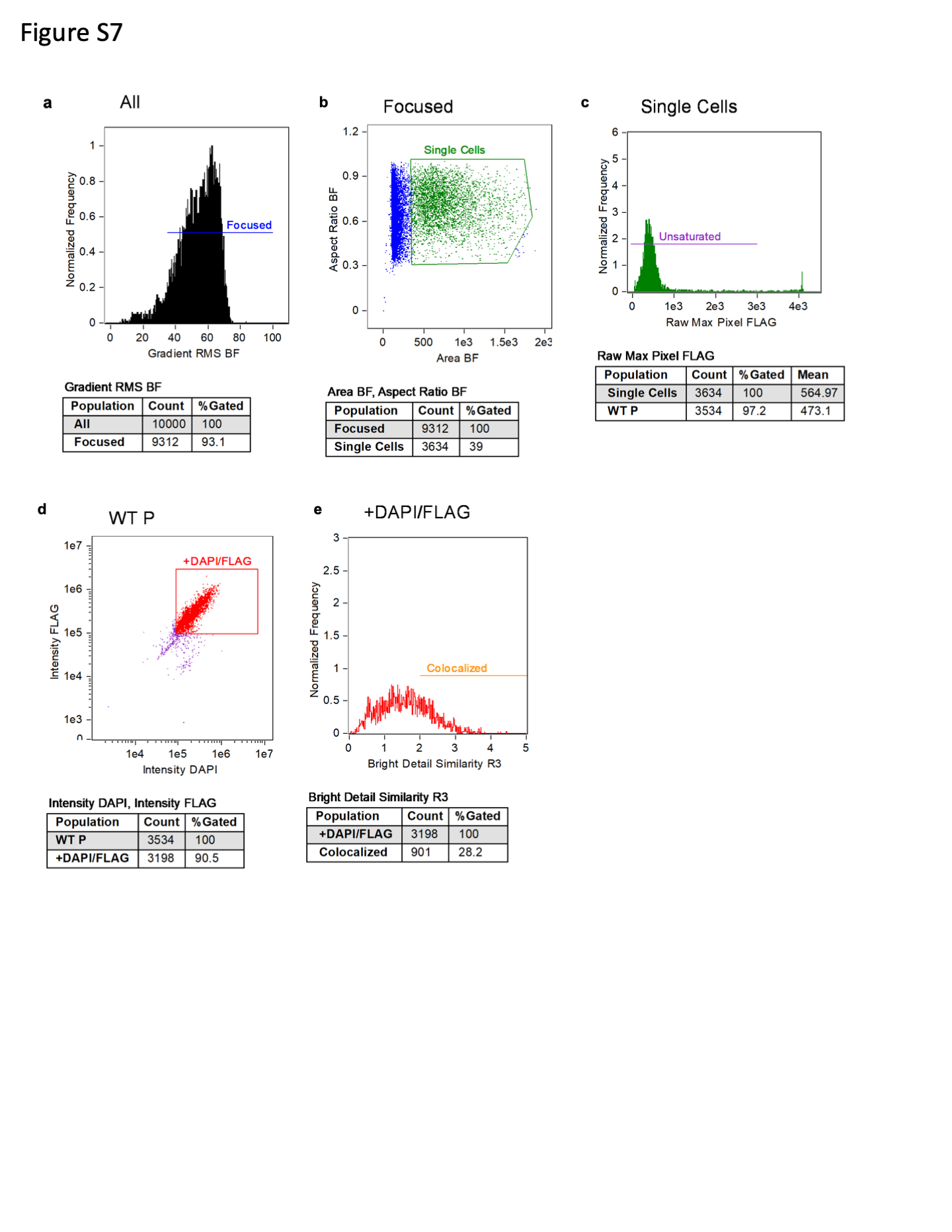

Supplement: S7 Fig — Shown is the gating strategy that was used to analyze imaging flow cytometry data, with the percentage of gated events, and number of gated events, shown below each plot. This example illustrates the gating of the wild-type sample shown in Fig 7. 10,000 events were collected. a, In-focus events were gated using a gradient of brightfield (BF). b, Events gated in panel a were refined to remove images containing more than one amoeba, by examining the area and the aspect ratio of the object(s) masked in the brightfield image. c, Events gated in panel b were refined to remove images that contained saturation in the FLAG channel (channel 11), by examining the raw max pixel intensity (i.e., the intensity of the brightest pixel in the image). d, Events gated in panel c as “unsaturated” were further examined to identify images that contained both FLAG and DAPI signal, by examining the intensity of the object masked in the DAPI and FLAG channels. e, Events gated in panel d as “+DAPI/FLAG” were further examined to characterize the degree of colocalization (bright detail similarity) between the DAPI and FLAG signals. (TIFF) [file ppat.1010088.s007.tiff]

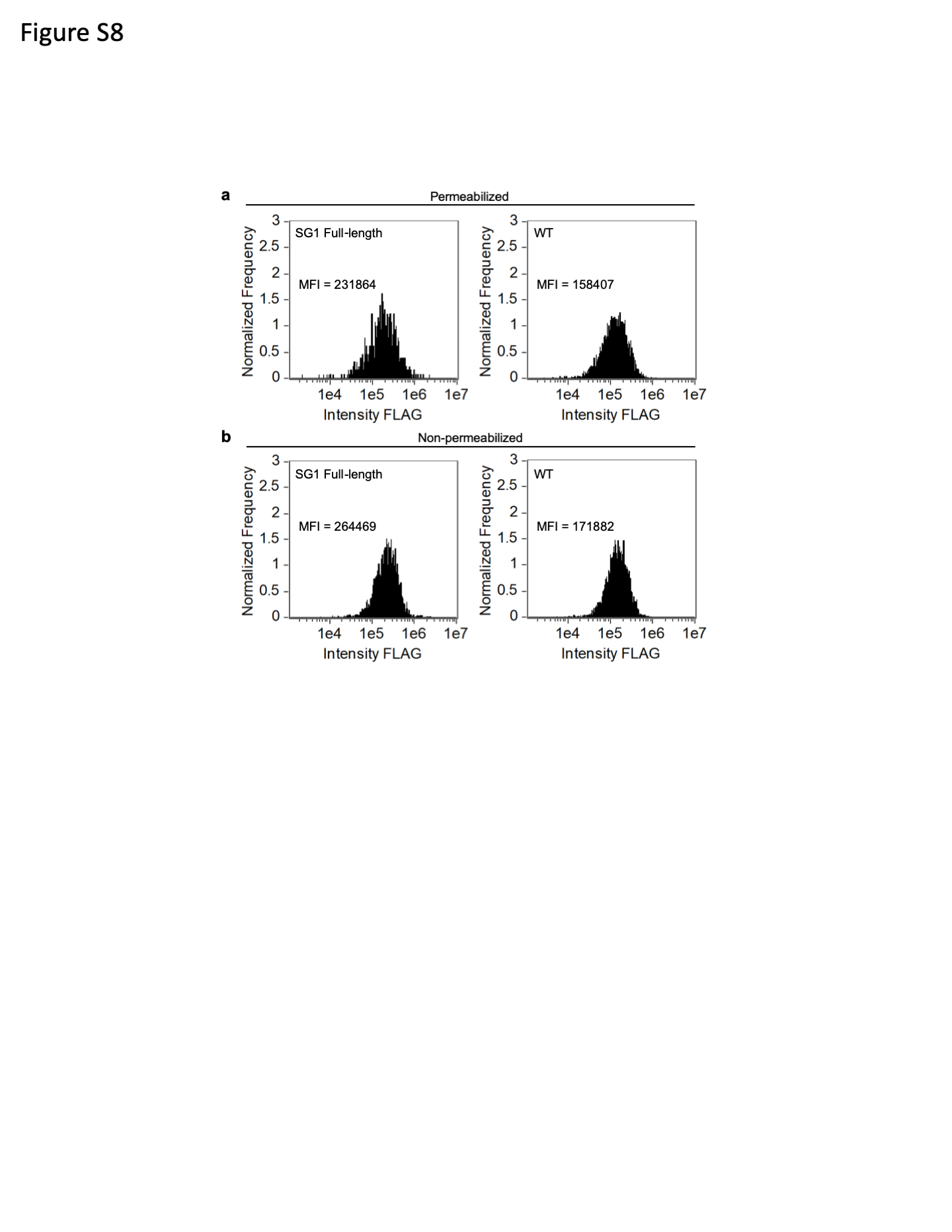

Supplement: S8 Fig — Amoebae were stably transfected with a plasmid for expression of full-length FLAG-tagged SG1. Immunofluorescence was used to determine the localization of SG1 in heterogeneous transfectants and imaging flow cytometry was used for analysis. The mean fluorescence intensity (MFI) is indicated on each histogram. a, Intensity of FLAG staining in FLAG-SG1 transfectants (left panel) and control staining of wild-type cells (right panel). Samples were permeabilized prior to antibody staining. b, Intensity of FLAG staining in FLAG-SG1 transfectants (left panel) and control staining of wild-type cells (right panel). Samples were not permeabilized prior to antibody staining. (TIFF) [file ppat.1010088.s008.tiff]

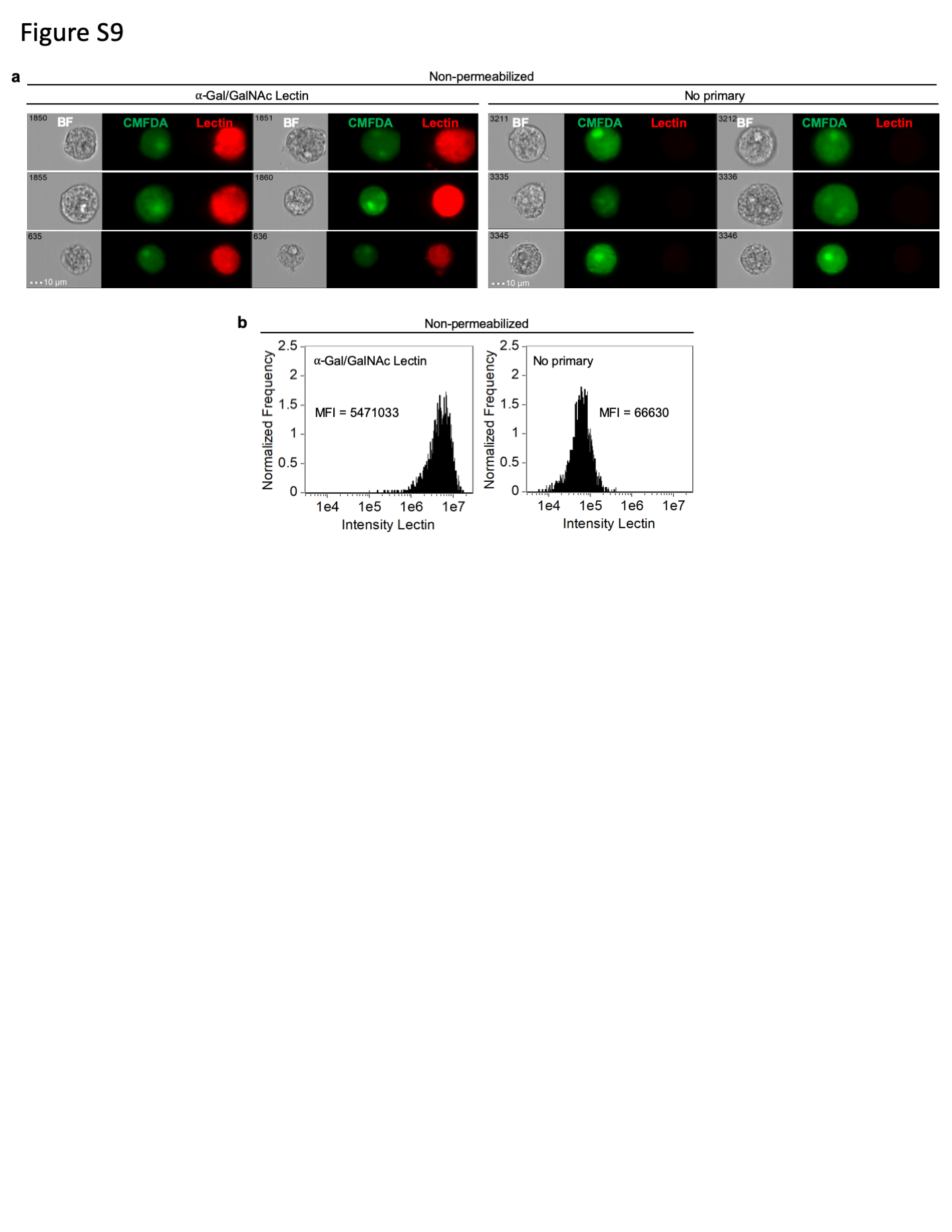

Supplement: S9 Fig — Amoebae were stably transfected with a vector control plasmid (pEhEx) and stained with the cytoplasmic dye CMFDA. Immunofluorescence was used to determine the localization of the endogenous Gal/GalNAc lectin, and imaging flow cytometry was used for analysis. a, Localization of the Gal/GalNAc lectin (left panel) and control staining of samples in which primary antibody was omitted (right panel). Samples were not permeabilized prior to antibody staining. Six random cells are shown for each condition. Shown from left to right are bright field images (BF), CMFDA staining (CMFDA, green), and mouse anti-Gal/GalNAc antibody staining (Lectin, red). The numbers in the BF images indicate the object/image number. b, Histograms showing the intensity of the Gal/GalNAc lectin staining (left panel) or control staining of samples in which primary antibody was omitted (right panel). The mean fluorescence intensity (MFI) is indicated on each histogram. (TIFF) [file ppat.1010088.s009.tiff]

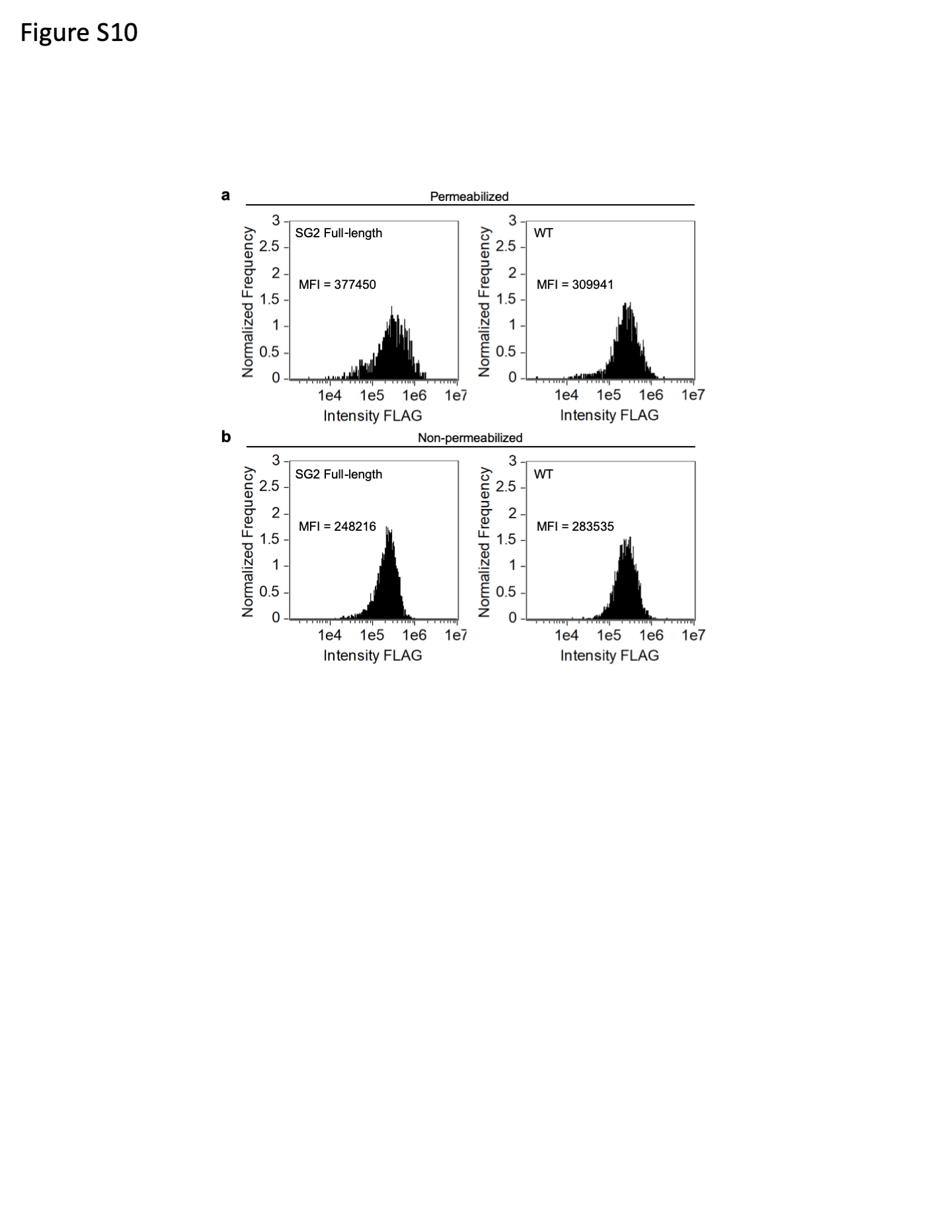

Supplement: S10 Fig — Amoebae were stably transfected with a plasmid for expression of full-length FLAG-tagged SG2. Immunofluorescence was used to determine the localization of SG2 in heterogeneous transfectants and imaging flow cytometry was used for analysis. The mean fluorescence intensity (MFI) is indicated on each histogram. a, Intensity of FLAG staining in FLAG-SG2 transfectants (left panel) and control staining of wild-type cells (right panel). Samples were permeabilized prior to antibody staining. b, Intensity of FLAG staining in FLAG-SG2 transfectants (left panel) and control staining of wild-type cells (right panel). Samples were not permeabilized prior to antibody staining. (TIFF) [file ppat.1010088.s010.tiff]

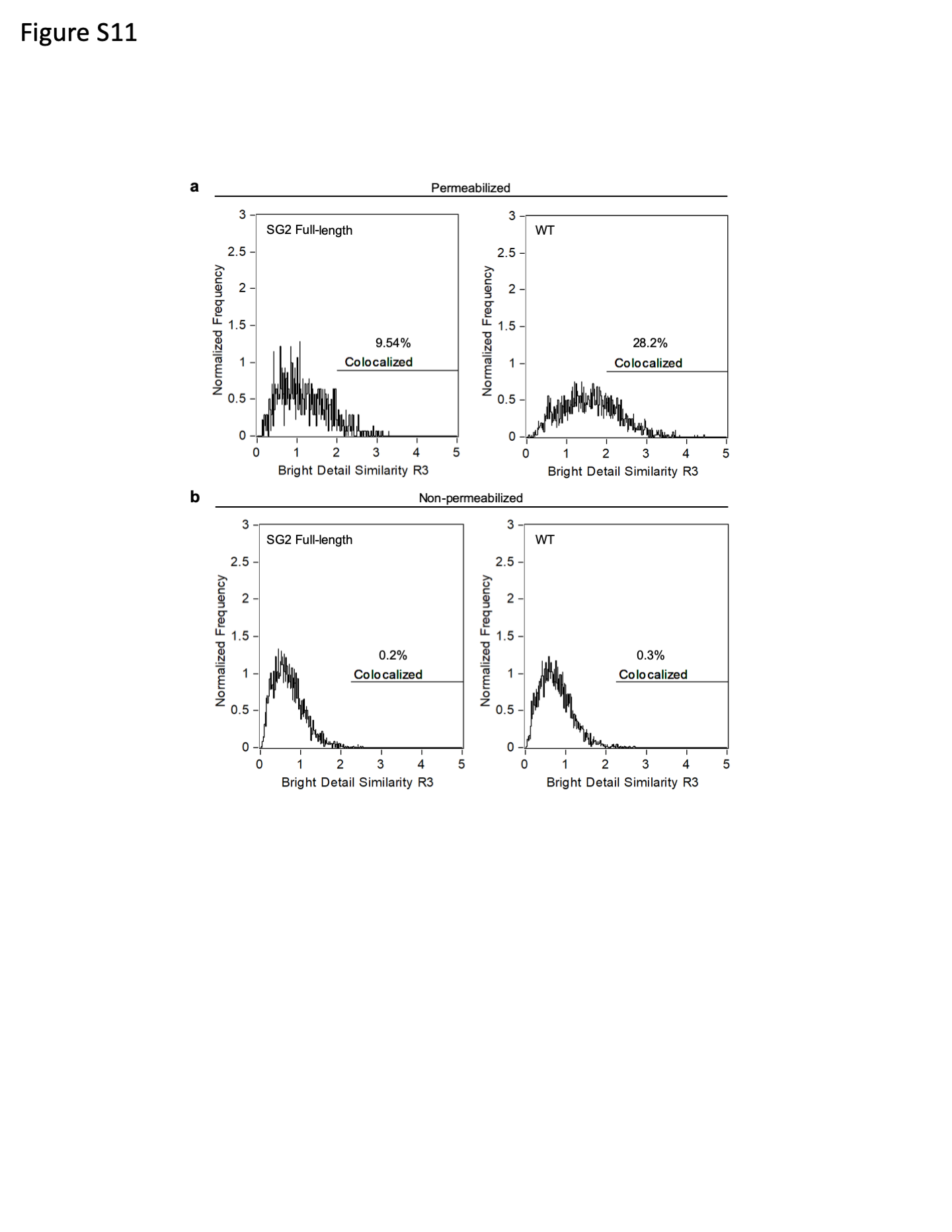

Supplement: S11 Fig — Nuclear cross-reactivity of the mouse anti-FLAG antibody was not seen in non-permeabilized cells, supporting that the sample preparation steps did not inadvertently permeabilize samples, and further supporting the surface staining patterns seen in non-permeabilized FLAG-SG1 cells. Colocalization analysis of FLAG and DAPI staining is shown. The percentage of DAPI+/FLAG+ images with colocalization of the two markers are indicated. Amoebae were stably transfected with a plasmid for expression of full-length FLAG-tagged SG2, or wild-type cells were analyzed as a control. a, Colocalization analysis of FLAG and DAPI staining in FLAG-SG2 transfectants (left panel) or wild-type cells (right panel). Samples were permeabilized prior to antibody staining. b, Colocalization analysis of FLAG and DAPI staining in FLAG-SG2 transfectants (left panel) or wild-type cells (right panel). Samples were not permeabilized prior to antibody staining. (TIFF) [file ppat.1010088.s011.tiff]

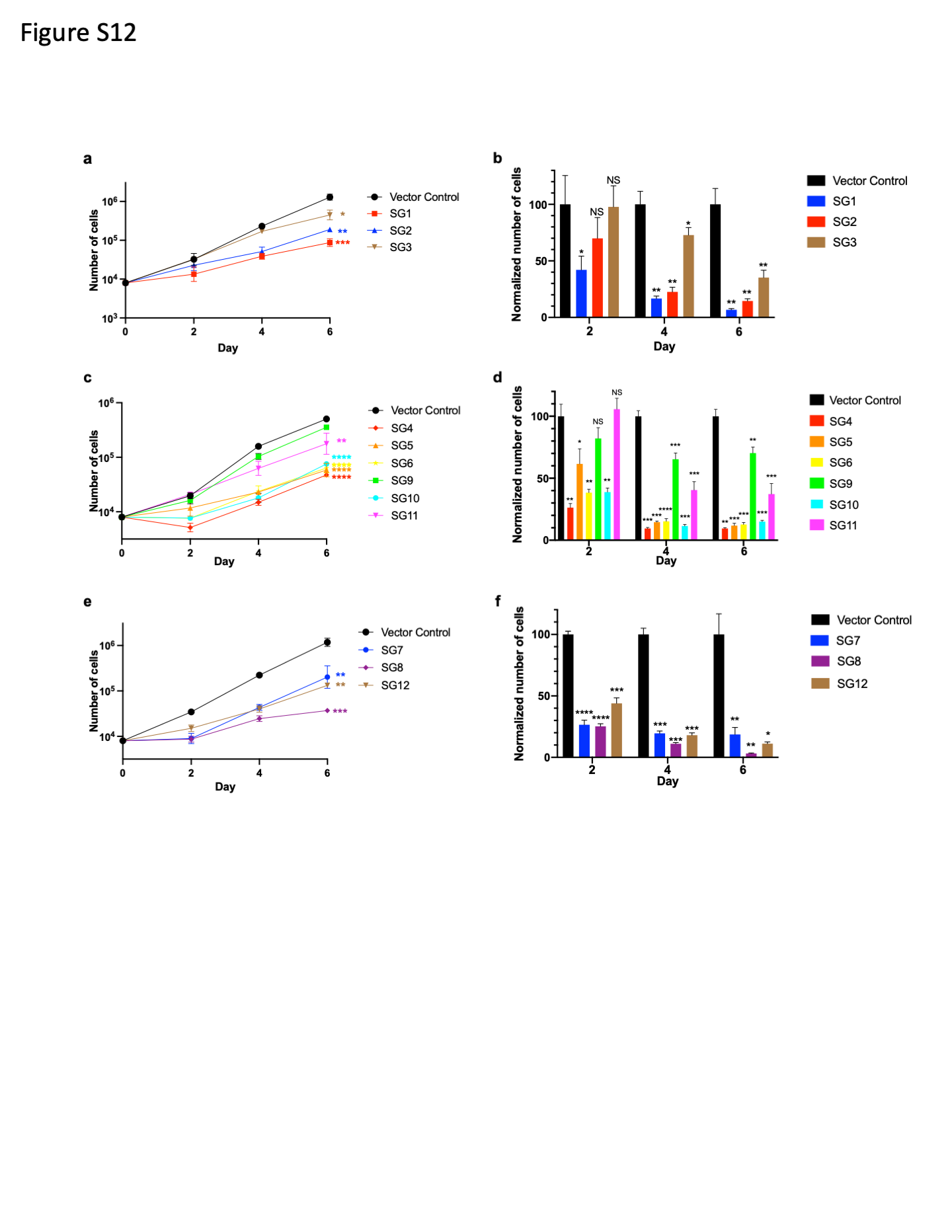

Supplement: S12 Fig — Growth analysis of mutants SG1 –SG12. a, c, e, Growth analysis demonstrates that selected clonal knockdown mutant lines have significant growth defects compared to vector control transfectants. b, d, f, Growth was normalized and compared to vector control transfectants on each day. Mutants exhibited statistically significant growth defects relative to vector control transfectants. (TIFF) [file ppat.1010088.s012.tiff]

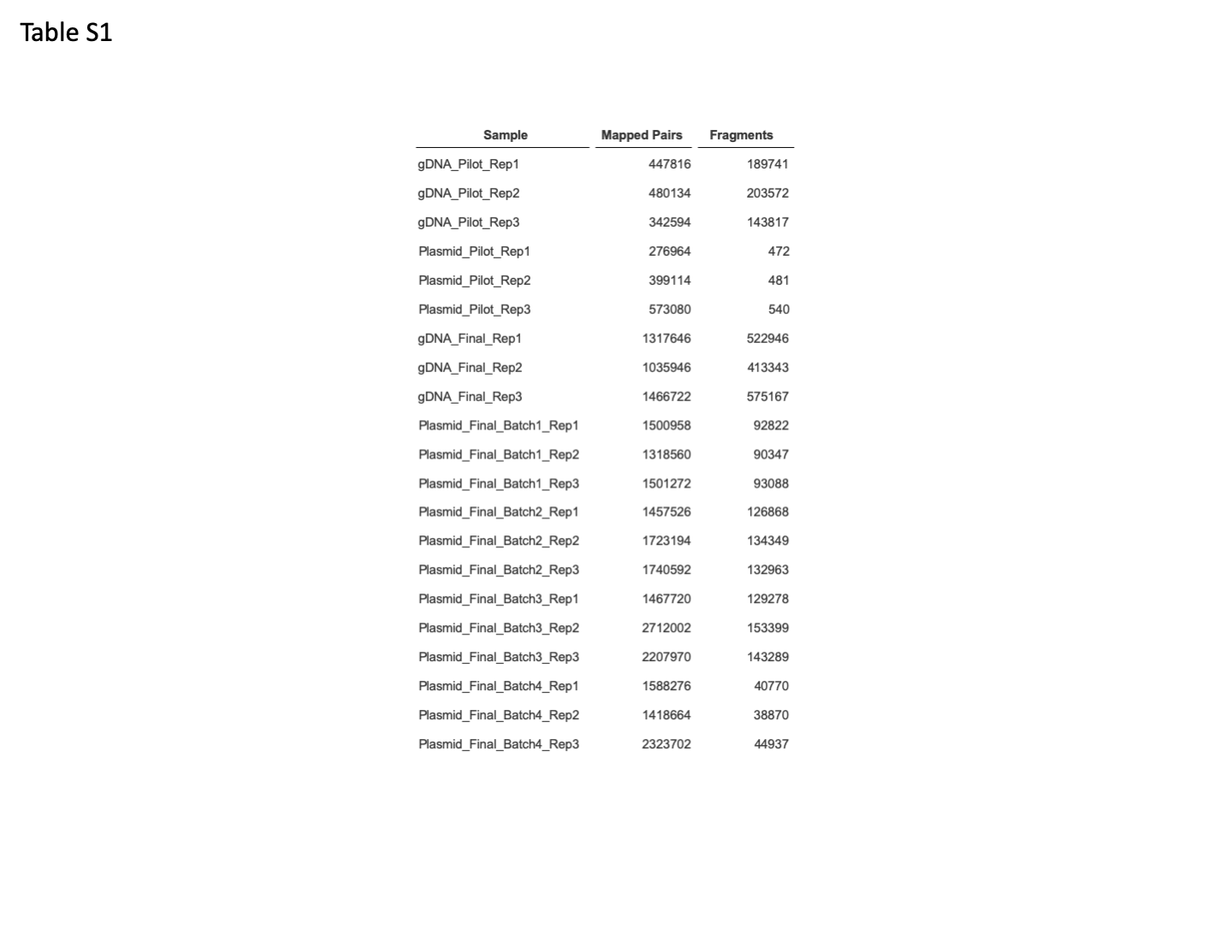

Supplement: S1 Table — Total number of paired reads mapping to the same contig (mappedPairs) and the number of fragments inferred from these mapped pairs for each sample. Reads were mapped with bwa mem, and processed from the resulting SAM formatted file using a custom Python script. Three sequencing replicates were performed for pilot gDNA samples and three sequencing replicates were performed for pilot plasmid samples. Three sequencing replicates were performed for final gDNA samples and three sequencing replicates were performed for each of four batches of final plasmid samples. (TIF) [file ppat.1010088.s013.tif]

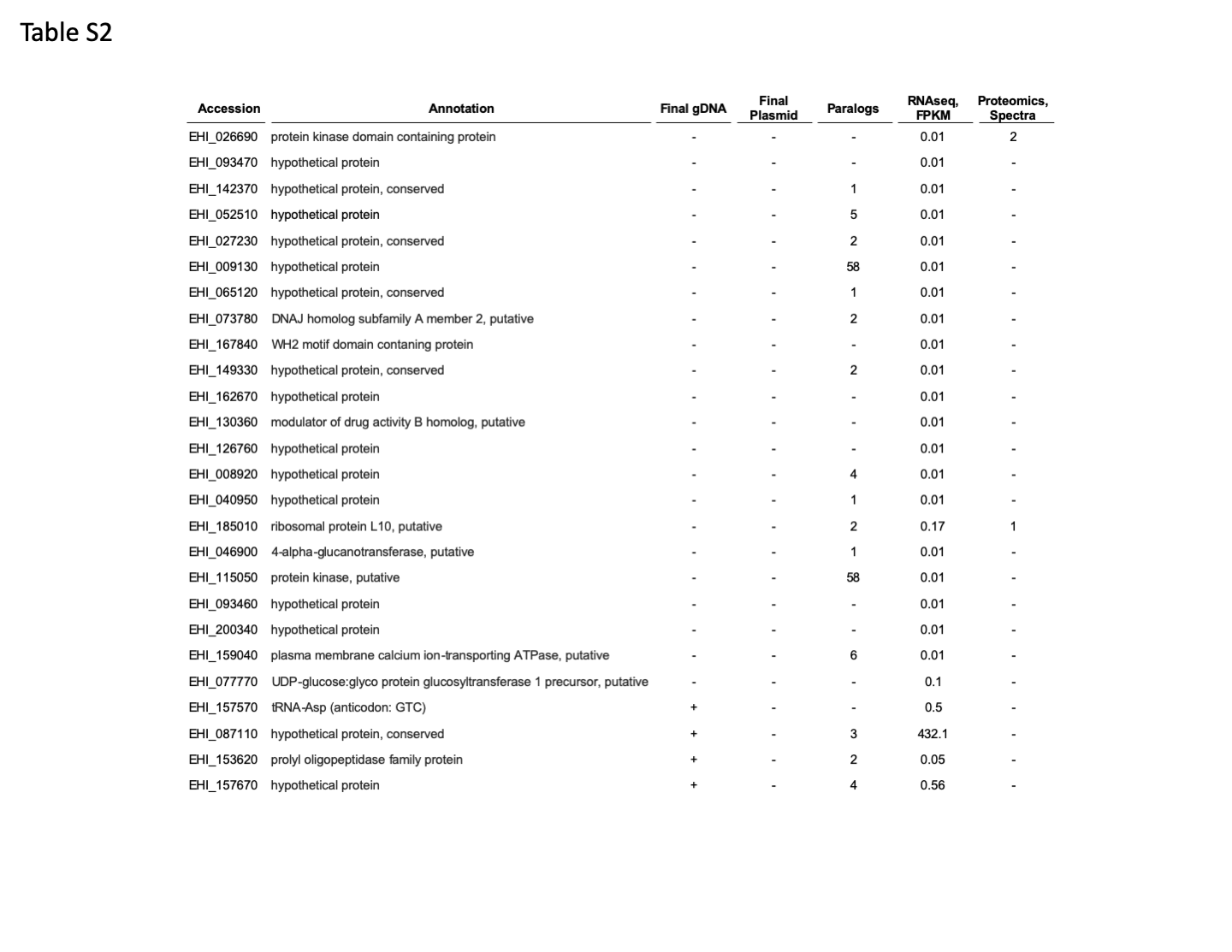

Supplement: S2 Table — Available empirical expression data for genes that were missing in the final plasmid library (genes that are represented in blue in S5 Fig). Accession numbers, annotation information, and the number of paralogs in the reference genome are indicated. Presence (+) or absence (-) in gDNA and plasmid datasets is shown. Gene expression from available RNAseq datasets [45,46] is indicated, as the FPKM + 1 value. The highest value for FPKM + 1 for each gene is shown. The total number of spectra that have been identified in proteomics studies is indicated. (TIF) [file ppat.1010088.s014.tif]

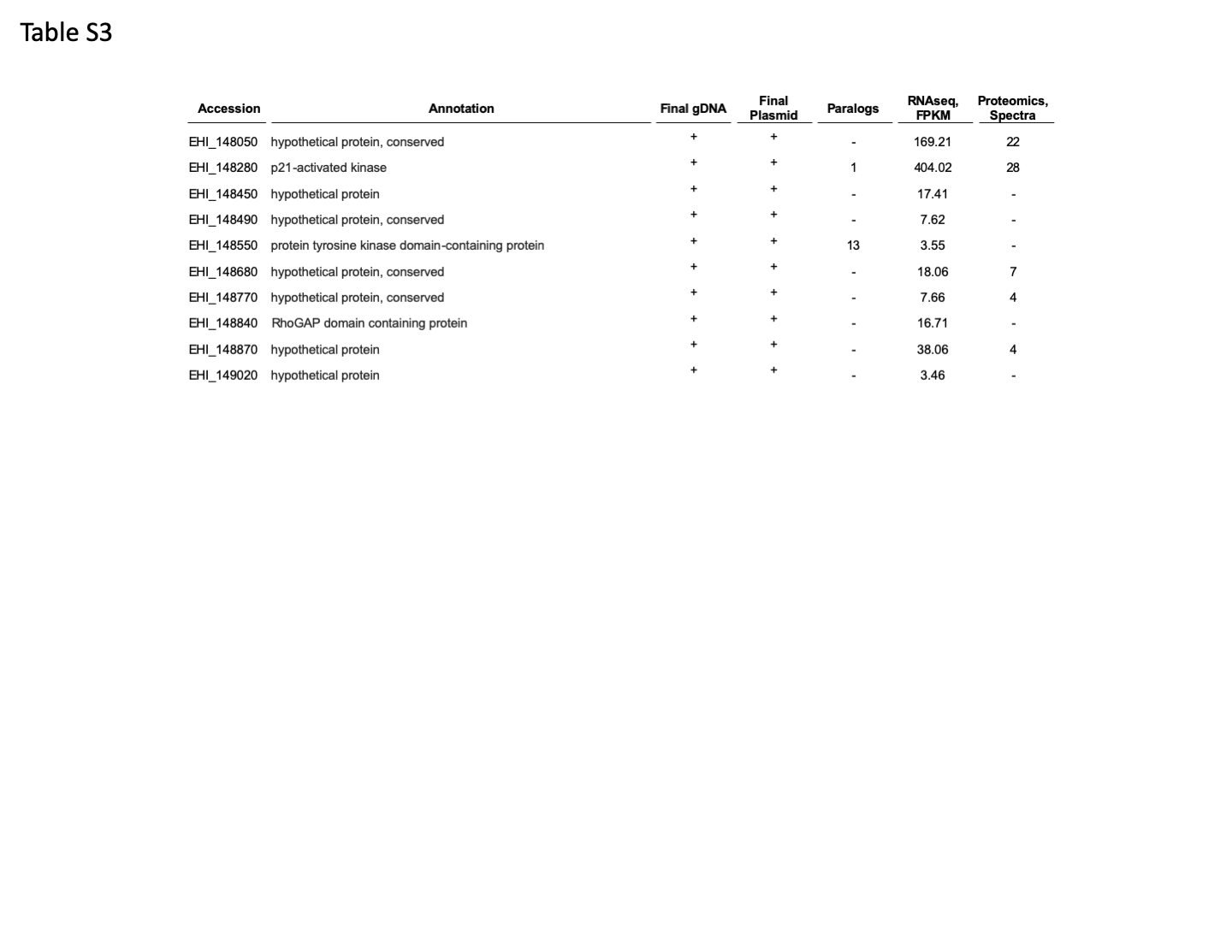

Supplement: S3 Table — To provide a frame of reference for the expression data in S2 Table, shown are the available empirical expression data for 10 random, representative genes that were present in the final plasmid library. Accession numbers, annotation information, and the number of paralogs in the reference genome are indicated. Presence (+) or absence (-) in gDNA and plasmid datasets is shown. Gene expression from available RNAseq datasets [45,46] is indicated, as the FPKM + 1 value. The highest value for FPKM + 1 for each gene is shown. The total number of spectra that have been identified in proteomics studies is indicated. (TIF) [file ppat.1010088.s015.tif]

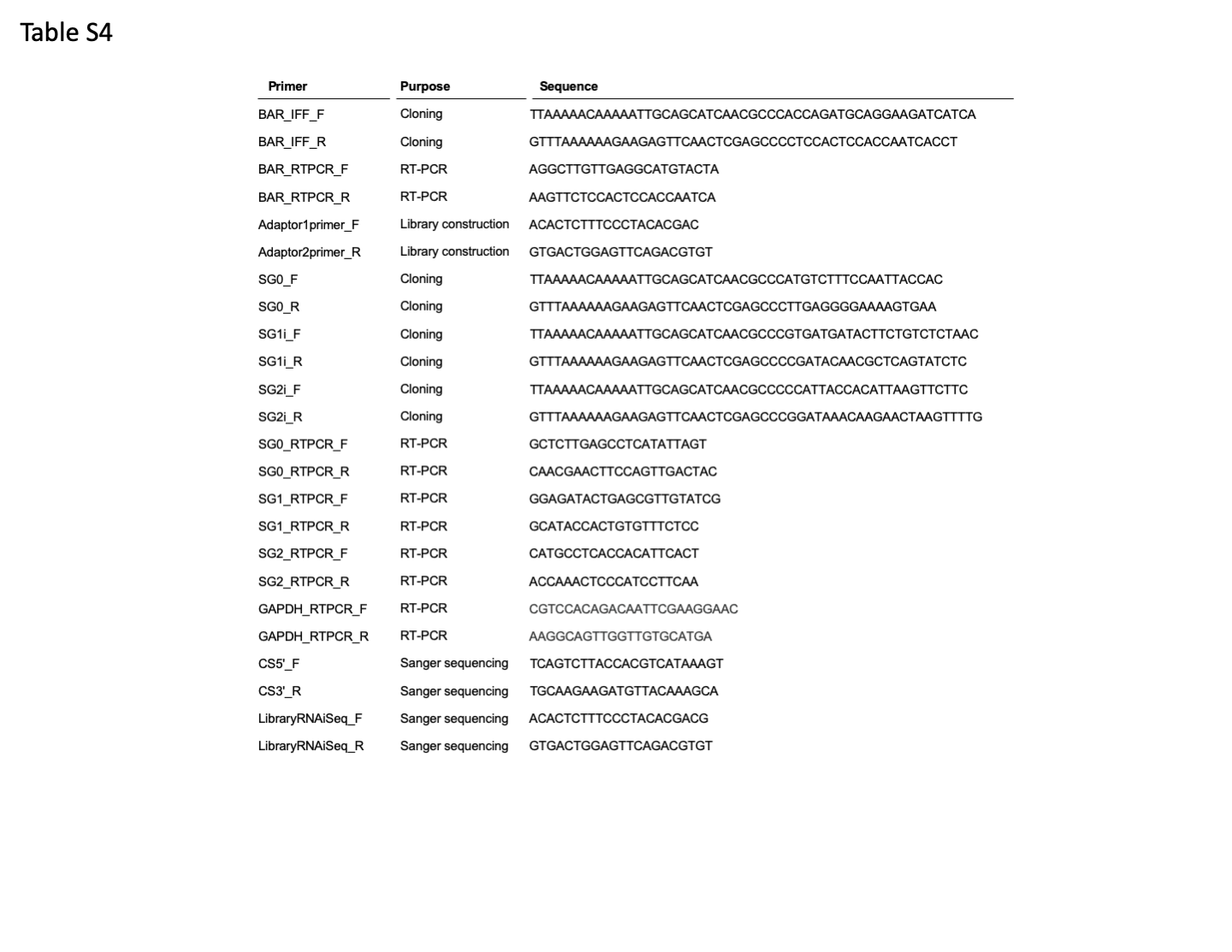

Supplement: S4 Table — Sequences of the primers used to generate and sequence plasmids, perform RT-PCR, and create the plasmid RNAi library. (TIF) [file ppat.1010088.s016.tif]
