# Supplementary material for: Establishment of quantitative RNAi-based forward genetics in Entamoeba histolytica and identification of genes required for growth
Source: PLoS Pathog. 2021 Nov 29;17(11):e1010088. doi: 10.1371/journal.ppat.1010088 (PMC8716031; doi:10.1371/journal.ppat.1010088)
Supplement: Attachment — Submitted filename: Response to Reviewers.docx [file ppat.1010088.s017.docx]

**RESPONSE TO REFEREES**

In the revised work, we have changed the manuscript to add insight into the biological function of two of the identified slow growth genes, and to address other critiques by the reviewers. **There are three entirely new figures (Fig. 6, 7, and S8).** The major changes are underlined in the manuscript text.

***Editor’s comments:*** *One approach to move forward would be for your group to follow up on one of the gene hits and garner more insight into its biological function. This would ideally be a gene that does not have a function that would otherwise be obvious and would thus demonstrate both the application of this technique and also the ability to garner novel biological insights using this RNAi screen.*

**Response:** We are grateful to the editor for considering the manuscript and for the constructive feedback. In the revision, we focused on garnering biological insight into two of the identified gene hits. While the localization of neither of the proteins targeted in SG1 nor SG2 mutants could be predicted by sequence analysis, **we showed experimentally that SG1 is both surface-localized and has a punctate cytoplasmic localization, while SG2 is localized throughout the cytoplasm (New Figures 6a – 6b and 7).** We also overexpressed full-length and truncation mutant proteins, and assayed for impacts on amoebic growth. **Overexpression of SG1 led to increased growth, while expression of a truncation mutant did not lead to increased growth, and thus aided in defining functional domains in this protein (New Figure 6d).** The increased growth rate of SG1 overexpressors was also reflected by a **quantitative increase in DAPI staining intensity (New Figure 6c),** as measured by imaging flow cytometry.

**PART I - SUMMARY**

***Reviewer #1:*** *The strength of this study is the development of forward genetics in Entamoeba. RNA interference using fragments of genomic DNA was used to identify genes whose knockdown resulted in slower growth. This is not only novel, but highly significant as it opens up the use of genetics to study this medically important and experimentally understudied parasite. The work is rigorously conducted and analyzed and the manuscript is clearly and modestly written.*

**Response:** We thank the reviewer and are honored to have received these encouraging comments.

***Reviewer #2:*** *In this study, the authors are the first to use forward genetics to construct and validate a genome-wide E. histolytica RNAi knockdown mutant library.  By RNAi screen, they have identified slow growing mutants and corresponding genes that affected cellular growth and metabolic pathways.  Growth retardation was confirmed experimentally using growth curves to characterize the mutants.  The molecular biology and genetic analysis were thorough and the screening procedures are well done and controlled.*

**Response:** We thank the reviewer for the positive comments on these studies.

***Reviewer #3:*** *The report by Bettadapur et al. describe the development of a novel RNAi-library for forward genetics screens in Entamoeba histolytica.  In particular, the authors used sheared gDNA to create a library of pTrigger plasmids. Inserts were flanked with sequencing adaptor sequences.  The library was transfected into E. histolytic cells.  The cells were cloned by limiting dilution and screened for growth defects.  Clones with growth defects were further characterized and confirmed to identify putative genes, which negatively impact growth when knocked down.  There is a paucity of genetic tools for this organism. Therefore, this is an important innovation for the field.  The study is particularly important because (1) it is the first RNAi library to be developed for this pathogen, and (2) the addition of Illumina adaptor sequences is novel and convenient. Despite these strengths, the authors have not convincingly shown the utility of this library because they did not
use it for screens on a mixed population of transformed parasites (please see comments in Part II below).*

**Response:** We thank the reviewer for the encouraging comments on the innovation of these studies. We will comment on mixed populations in Part II, below.

**PART II – MAJOR ISSUES**

***Reviewer #1:*** *The authors might consider cloning of E. histolytica in Petri dishes in soft agar which has been reported as an additional method to limiting dilution.*

**Response:** We thank the reviewer for the suggestion of cloning in soft agar plates. **We modified the text to describe cloning in soft agar plates (in lines 346 – 348), “We obtain clonal lines by limiting dilution, but another effective available method is cloning by isolating *E. histolytica* colonies on soft agar plates”.** We have relatively limited experience with cloning in soft agar plates, and are working to refine our technique. Cloning by limiting dilution in 96-well plates has been extremely effective in our hands, and is described in detail our manuscript recently submitted to *Current Protocols* (Suleiman, RL and Ralston, KS. Growth and genetic manipulation of *Entamoeba histolytica. Current Protocols*).

*Would it make sense to validate one or more of the genes identified as growth-related through a separate technique such as dominant negative or over-expression, and control for effects of RNA interference through expression of a scrambled RNAi? Perhaps this is unnecessary since the genome-wide library is in itself a control.*

**Response:** We agree and thank the reviewer for the suggestion. To validate a gene identified as growth-related, and to also gain a deeper understanding of domains, we overexpressed full-length and truncation mutant proteins, and assayed for impacts on amoebic growth. **Overexpression of SG1 led to increased growth, while expression of a truncation mutant did not lead to increased growth, and thus aided in defining functional domains in this protein (New Figure 6d).** The increased growth rate of SG1 overexpressors was also reflected by a **quantitative increase in DAPI staining intensity (New Figure 6c),** as measured by imaging flow cytometry.

***Reviewer #2:*** *1. In general, I share the author’s enthusiasm with this important discovery that has huge potential going forward.  However, the manuscript reads more like a methods paper with very little biological characterization.  Using the discovery as a stepping stone to elucidate important virulence genes in disease pathogenesis or with vaccine potential would more impact.*

**Response:** We thank the reviewer for their enthusiasm and for the feedback. In the revised manuscript, **we added new biological characterization of genes identified as hits in our genetic screen (New Figures 6, 7 and S8).** While the localization of neither of the proteins targeted in SG1 nor SG2 mutants could be predicted by sequence analysis, **we showed experimentally that SG1 is both surface-localized and has a punctate cytoplasmic localization, while SG2 is localized throughout the cytoplasm (New Figures 6a – 6b, 7).** To gain a deeper understanding of SG1 function, transfectants overexpressing the full-length protein or a C-terminal truncation were generated. **Overexpression of SG1 led to increased growth, while expression of a truncation mutant led to decreased growth, and thus aided in defining functional domains in this protein (New Figures 6c – 6d).** The increased growth rate of SG1 overexpressors was also reflected by a **quantitative increase in DAPI staining intensity (New Figure 6c),** as measured by imaging flow cytometry. Like the reviewer, we are enthusiastic about the new discoveries that are now possible through the use of our forward genetic library, but we believe that creating an RNAi library, using it in the first forward RNAi screen in *E. histolytica*, and biologically characterizing the hits of the screen already form a significant advance for the field. We believe that elucidating genes involved in disease pathogenesis or vaccine potential are beyond the scope of the current study.

*2. The only functional data is Figure 5 and the growth curves are not remarkable.  Considering a 4-day growth for E. histolytica prior to sub passage, the relevance of Day 6 growth is questionable.  These parasites usually crash in culture at Day 6.*

**Response:** We thank the reviewer for their critical evaluation of the growth curves. In the revision, we note that we now have additional functional data (i.e., localization, domain analysis, overexpression analysis). **We have also performed a new growth curve that spans five days (New Figure 6d).** It is important to recognize that the starting cell concentration is a critical parameter, and that by seeding cells at different starting concentrations, the time it takes for parasites to “crash” will vary. In our studies, cells were seeded at a very low concentration (8x10^3^ cells per culture tube) on Day Zero, and vector control transfectants were always included as a control sample. **Importantly, growth of vector control transfectants remained exponential through Day Six in all growth curves (Figures 5a, 5c, S7)**; thus there was no evidence of the cultures crashing due to reaching an excessive cell concentration in this timeframe. Moreover, **all of the slow growth mutants exhibited a statistically significant growth defect by Day Four (or earlier).** We also point out that the growth defects we reported in Table 2 are all from Day Four. Finally, the reviewer stated that the growth curves are “not remarkable,” so we emphasize that **the majority of slow growth mutants that we isolated in these studies have growth defects that are more pronounced than mutants knocked down for expression of a known essential gene** (superoxide dismutase, a target of the drug metronidazole [PMID: 17676992]), that we used as a control for slow growth (Fig. 5a).

*3. The relevance of the defective growth mutants to the biology of the parasite in colonization or disease should be tested.*

**Response:** Like the reviewer, we are enthusiastic about the new discoveries that are now possible through the use of this forward genetic library, but we believe that studying the phenotypes of slow growth mutants in colonization or disease is beyond the scope of the current study. Moreover, since the mutants have known growth defects *in vitro*, they would be expected to have growth defects *in vivo,* and to thus exhibit defects in colonization or pathogenesis. Therefore, it would likely be challenging to glean new, meaningful information from *in vivo* studies.

*3. The data in Figure 5e should be compared to WT cells and not between the mutants.  Even then, the significance of Day 6 growth is out of range as peak log phase usually occur around Day 3 in culture.*

**Response:** We thank the reviewer for their comments on Figure 5e. In these data, the mutants were indeed compared to vector control transfectants (which grow at WT rates); we did not perform a comparison between the mutants. In the revision, **we added more explanation to make this more clear (in line 959), “Growth values for each mutant were normalized to vector control transfectants”.** With regards to the comments about the significance of Day Six growth, we again emphasize (1) that the concentration of cells on a particular day is dependent on the concentration at which they were seeded; (2) that all slow growth mutants exhibited statistically significant slow growth by Day Four (or earlier); (3) that the growth defects we reported in Table 2 are all from Day Four; and (4) that the growth of vector control transfectants remained exponential through Day Six in all experiments. **We have also performed a new growth curve that spans five days (New Figure 6d).**

***Reviewer #3:*** *1. It is somewhat surprising that the authors chose “inhibition of growth” as the phenotype to illustrate the utility of the RNAi library. Because slow growers would be overtaken in a mixed population, a screen for such can only be done on clones.  Thus, the utility of the library for high throughput screens on a mixed population of transformed parasites has not demonstrated and the report may be premature. The significance of the report would be strengthened by demonstrating the use of the library in a high throughput screen.*

**Response:** We thank the reviewer for their critique. **Indeed, the design of our RNAi library means that mutants that are outcompeted (i.e., diminished in quantity or even “missing”) in a mixed population can absolutely be identified.** Our plasmid RNAi library includes Illumina sequencing adaptors that flank the RNAi library inserts. This allows for Illumina deep sequencing analysis to quantitatively identify mutants that are over- or under-represented, as well as their relative abundance. Mutants that are outcompeted in a mixed population can be identified using such an approach, by comparing the representation of RNAi library mutants immediately after stable transfection, and at various time points following transfection, to track the mutants that diminish in representation over time. It is also possible to compare the overall makeup of the plasmid library prior to transfection, versus immediately after stable transfection, to identify essential genes, for which viable mutants are never seen in the transfected population. Our approach builds on many examples of screens in parasites that have similarly used next generation sequencing to identify mutants that are missing or lost over time. **This is described in lines 120 – 123: “More recent studies used next generation sequencing on non-clonal mutant populations in order to identify mutants and quantify their relative abundance [32-39]. The latter approaches are useful because they can quantitatively identify mutants that are enriched by selection, and mutants that are less abundant or are lost after selection.”** We support the reviewer’s enthusiasm for performing a high-throughput screen, in contrast to the medium-throughput screen performed here, but given the current limited tractability of *E. histolytica,* we believe that reporting the establishment of forward genetics and the proof of concept screen for slow growth mutants form a solid first chapter to these types of studies in this organism*.*

2. It is not clear how the authors initially identified slow growing clones.  The authors state in the methods: “clonal lines that visually appeared to have slow growth.” Was this done by microscopy or simple visualization of the attached cells by observing the plate directly?

**Response:** We thank the reviewer for pointing this out. Clonal lines that appeared to have slow growth were identified by using microscopy. To improve clarity, **we added additional text (in lines 589 – 590), as follows: “For identification of mutants with growth defects, clonal lines that visually appeared to have slow growth when examined using microscopy were selected for further analysis.”**

**PART III – MINOR ISSUES**

***Reviewer #1:*** *(No Response)*

***Reviewer #2:*** *1. The introduction is 5 pages long.  This can be reduced to 2-3 pages.*

**Response:** We thank the reviewer for this suggestion. **In the revised article, the introduction has been reduced to 3 pages.**

*2. The text in most of the computer generated graphs (figures) were difficult to read and should be redone with font size that are visible in power point.*

**Response:** We thank the reviewer for pointing out the small font size. **In the revised article, we enlarged the font size in all computer generated figures.**

***Reviewer #3:*** *3. The authors cite unpublished data (Line 351).  This may not be allowed by PLoS Pathogens.*

**Response:** We thank the reviewer for noting this. **In the revised manuscript, we removed the citation of unpublished data, and replaced it with a new citation (in Line 346).** The data we were referring to are now described in detail in a manuscript recently submitted to *Current Protocols* (Suleiman, RL and Ralston, KS. Growth and genetic manipulation of *Entamoeba histolytica. Current Protocols*).
