# Supplementary material for: Establishment of quantitative RNAi-based forward genetics in Entamoeba histolytica and identification of genes required for growth
Source: PLoS Pathog. 2021 Nov 29;17(11):e1010088. doi: 10.1371/journal.ppat.1010088 (PMC8716031; doi:10.1371/journal.ppat.1010088)
Supplement: Attachment — Submitted filename: Response to Reviewers.docx [file ppat.1010088.s018.docx]

**RESPONSE TO REFEREES**

In the revised work, we have modified the text and performed new experimentation to address the minor critiques of all reviewers. **There are four entirely new figures (Fig. S8, S9, S10, S11), and one new figure panel (Fig. 7b).** The major changes are underlined in the manuscript text.

***Editor’s comments:*** *You will note that one reviewer requested extensive major revisions including expansion of the study. We understand that this is beyond the scope of this manuscript and do not expect those experiments to be done. Despite these concerns we agree with Reviewers 1 and 2 that the impact of this work is substantial and important to move the field forward. In a revised manuscript, please address the minor comments by all reviewers, either experimentally or in the response/revised manuscript.*

**Response:** We are grateful to the editor for considering the manuscript and for the constructive feedback. In the revision, **we focused on addressing the minor comments of all reviewers, both by revising the text and by adding new figures.**

**PART I - SUMMARY**

***Reviewer #1:*** *In this study, Bettadapur and colleagues describe the first forward-genetics RNAi screen in the human protozoan pathogen, Entamoeba histolytica. This pathogen is a globally-important causative agent of dysentery and liver abscess. There is no vaccine and currently available drugs are fraught with side effects. Thus, any molecular and or cellular insight into this pathogen may reveal new targets for therapy. This study provides a new tool for the community of researchers to identify such targets. The strengths of the study are its novelty and robust analyses of the RNAi libraries. The authors have nicely responded to the previous critiques.*

**Response:** We thank the reviewer for the encouraging comments on the novelty and robustness of the work.

***Reviewer #2:*** *Bettadapur et al generate a library of gDNA fragments flanked by an RNAi trigger in order to establish a forward genetics screening system for E. Histolytica. They use this system to screen 80 transgenic cell lines and identified 12 Slow Growth (SG) mutants. They sequenced the inserts to identify the targeted genes and learned that the orientation of the gDNA fragment has no impact on the effectiveness of knockdown. Toward characterizing SG1 and SG2 mutants they localized these proteins.*

**Response:** We thank the reviewer for their comments on the work.

***Reviewer #3:*** *This paper from Bettadapur, et al, takes a very creative approach to establish the first forward genetic gene-silencing system for Entamoeba histolytica that is suitable for conducting genome-wide screens. The authors do a great job describing the approach, building and characterizing a plasmid library, and successfully completing a pilot screen that validates the method. The work appears to have been done to a high standard, and the figures and writing are excellent. As noted on the prior reviews, there is limited biological follow up on "hits" from the pilot screen, although this appears to have been augmented by confirming phenotypes and looking at localization for two of the silenced genes. I did not review the original version of this manuscript and I have to say that I think the importance of the paper is in description and validation of the method. The reality is that this type of method has been sorely needed and has the potential to jump start Entamoeba histolytica research--the lack of magnificent new biological insights from the pilot screen is no surprise, and, given the time and resources that would be needed to conduct a whole-genome screen with thorough follow up for a more interesting phenotype, I personally think it would do the Entamoeba research community (and perhaps people affected by amebiasis) a disservice to insist on more work with a resultant delay in publication. I have minor comments that, if addressed, would strengthen the manuscript.*

**Response:** We thank the reviewer and are honored to have received these positive comments.

**PART II – MAJOR ISSUES**

***Reviewer #1:*** *No major issues.*

**Response:** We thank the reviewer for their comment.

***Reviewer #2:*** *1. The authors constructed a high-quality library, but the utility of this library has not been established. On Line 505 the authors state that complete genome coverage requires 36,354 plasmids. If each isolated transgenic parasite caries a unique plasmid, then the 80 isolated parasites screened in this manuscript represents about 0.22% genome coverage. Screening 80 colonies does not meet the bar for a forward genetics screening system. Consider that knockdown of 80 genes is still well within the range of what is possible for a directed screen, the authors need to devise a means to screen at least several thousand inserts before they can conclude they have a game changing forward genetics system.*

**Response:** We thank the reviewer for their critique. We support the reviewer’s enthusiasm for performing a high-throughput screen, in contrast to the medium-throughput screen performed here, but given the current limited tractability of *E. histolytica,* we believe that reporting the establishment of forward genetics and the proof of concept screen for slow growth mutants form a solid first chapter to these types of studies in this organism*.*

*2. Why report generating a complete library if the performed screen was from the pilot library? Given that only 80 strains were screened the pilot library already contains far more unique plasmids. The authors need to work on a method to increase the throughput of screening.*

**Response:** We appreciate the reviewer’s criticism. We report the development of the complete library in order to establish the tool for the *E. histolytica* community, and we look forward to sharing it with the community, so that we and others can enjoy its use in enabling future high-throughput screening.

*3. Slow growth is not a particularly informative phenotype. I imagine the authors have some plan to use this screening strategy to probe a biological process of interest. A demonstration that the screening system could be used to identify mutants that are defective in a cellular process of interest would be more meaningful and impactful.*

**Response:** We acknowledge the reviewer’s lack of enthusiasm for the slow growth phenotype. Notably, the slow growth phenotype begins to provide functional information to genes for which existing annotation does not inform understanding of protein function. All of the annotated gene hits in our screen had homologues in the related human-infectious *Entamoeba* species *E. dispar, E. nuttali* and *E. moshkovskii*. Thus, this type of screen may be informative in identifying potential drug targets that are conserved among human-infectious *Entamoeba* species. We look forward to sharing the RNAi library with the community, so that we and others can use it in future screens for other biological processes of interest.

***Reviewer #3:*** *None.*

**Response:** We thank the reviewer for their comment.

**PART III – MINOR ISSUES**

***Reviewer #1:*** *1. The conclusion that the protein encoded by EHI_194310 is on the cell surface is not well-supported by the immunofluorescence data. There is a “haze” of staining on the “unpermeabilized” cells, but it is difficult to see. Even fixation with paraformaldehyde will cause some permeabilization. Thus, it possible that the haze is simply some of the anti-FLAG antibody getting into slightly permeabilized cells. This would be particularly true if the expression of the FLAG-tagged version of the protein was high. The authors state that they did not observe such surface staining in the EHI_007010 overexpressing cell line, but they did not show the data. This reviewer assumes that such a conclusion was drawn because no haze was observed whatsoever. Perhaps providing the EHI-007010 staining data and showing that it is different from the EHI_194310 staining data might be more convincing. Comparing the image analyses for both staining experiments might also help illustrate the contrast.*

**Response:** We thank the reviewer for pointing this out, and **we have added new Figures 7b, S8, S9, S10 and S11 to address this critique.**

Additional data that are now added to the manuscript support that FLAG-SG1 is detected in non-permeabilized cells, but FLAG-SG2 is not. Imagery from non-permeabilized FLAG-SG2 (EHI_007010) expressing cells **(New Fig. 7b), demonstrates that staining is not detected in non-permeabilized FLAG-SG2 expressers.** Image analysis **(New Figures S8b, S10b) shows that FLAG staining is above background in non-permeabilized FLAG-SG1 cells, while it is not above background in non-permeabilized FLAG-SG2 cells.**

We found that the anti-FLAG antibody cross-reacts with the nucleus. Importantly, this **nuclear cross-reactivity is not seen in non-permeabilized cells (New Figure S11), supporting that the non-permeabilized cells are not permeable.**

Immunofluorescence analysis of the amoeba surface **Gal/GalNAc lectin in non-permeabilized cells has been added (New Figure S9), which shows an example of surface staining with a known surface protein.**

Finally, we have also **added image analysis data for all experiments (New Figures S8, S9, S10), which shows that FLAG-SG1 and FLAG-SG2 are both expressed at a very low level,** which contrasts with the high intensity expression of the endogenous Gal/GalNAc lectin. Thus, the level of expression is unlikely to have created an artifact, as the reviewer suggests.

Together, the new figures support that non-permeabilized cells are not permeable, that the staining pattern of FLAG-SG1 in non-permeabilized cells resembles the pattern of the Gal/GalNAc lectin, and that the low expression levels of FLAG-SG1 and FLAG-SG2 likely explain the relative “haziness” of the images.

*2. In the Introduction, the authors provide a nice review and timeline of the development of forward genetics and RNAi techniques for use in this parasite. Recently, a study showing proof of principle of a CRISPR/Cas9 system for use in Entamoeba (Kangussu-Marcolino et al., 2021). It would be fair to include this information/cite this study in the Introduction and/or Discussion.*

**Response:** We thank the reviewer for noting that the Kangussu-Marcolino article, which came out in the course of the revision of this manuscript, had not been cited. **This important new study is now described and cited in lines 90 – 92,** “Very recently, CRISPR/Cas9 has been established in *E. histolytica* (Kangussu-Marcolino, 2021, PMID: 33264648), providing an important new tool for future genetic manipulation of this organism.”

*3. The data in Figure 5 are not uniformly presented. In 5B, two of the panels present the RT data first (+ -), while the third panel presents the RT data second (- +). In 5D, RT data are presented second (- +). This is confusing to the reader*

**Response:** We agree and thank the reviewer for noticing this issue. Unfortunately, if we flip the image orientation so that the panels are consistent with regards to the order of the +/- RT data, then the SG2 panel will be flipped with regards to the order of the RNAi and WT samples. In light of this, we found it less confusing to have some of the +/- RT data flipped, than to have the mutant vs. control data flipped.

***Reviewer #2:*** *1. The characterization of SG1 and SG2 is not informative. I am not suggesting the authors do more because this is not my major issue with the work.*

**Response:** We thank the reviewer for their comment.

*2. The choice of Imaging flow cytometry is not justified. The authors could acquire much higher resolution images using a confocal with a high NA 60 or 63X objective with the advantage of being able to see how the protein localization changes at different focal planes within cells. It is not clear that SG1 is in the plasma membrane. It could be in the ER and be pushed up against the cell cortex. Consider using a plasma membrane stain or protein marker already known to be at the plasma membrane.*

**Response:** We thank the reviewer for the suggestion. Immunofluorescence analysis of the amoeba surface **Gal/GalNAc lectin in non-permeabilized cells has been added (New Figure S9), which shows an example of surface staining with a known surface protein.** The reviewer is correct that the ability to resolve fine details is higher with confocal microscopy than imaging flow cytometry. However, an important point is that the **sensitivity of imaging flow cytometry is higher than confocal microscopy**, meaning that fluorescent signal that appears weak on a confocal microscope can be detected readily on an imaging flow cytometer. **The image analysis that we have added to the revision (Figures S8, S9, S10) shows that the expression level of FLAG-SG1 and FLAG-SG2 is very low, and is dramatically different than the high intensity expression of the endogenous Gal/GalNAc lectin.** As the reviewer suggests, we tried imaging these cells on a spinning disk confocal microscope with a 63X objective (NA 1.4), but the fluorescence intensity was too dim to reliably analyze. Hence, we concluded that **the use of imaging flow cytometry is justified for sensitive detection of a dim signal, and for its ability to enable unbiased quantitative image analysis of thousands of images.**

*3. The authors chose to use 500 bp fragments in the library. Clearly this size fragment can work. Could the authors compare a 500 bp vs 1000 bp fragment for their BAR/SH3 control gene to give us some sense for how their choice to use 500bp fragments in their library might impact efficacy of silencing?*

**Response:** This is a good question, but **this has already been addressed in the literature (Khalil, et al., PMID: 26747561).** Using the trigger plasmid approach, a 537 bp fragment of EhRom1 was compared with a 993 bp fragment (i.e., the full coding sequence of the gene), and the authors concluded “The down-regulation obtained using a portion of the gene was comparable to the silencing obtained using the full-length gene” (Khalil, et al., PMID: 26747561).

We carefully weighed the fragment size when making the plasmid library, and **we actually used 500-900 bp fragments in the final library, not 500 bp as the reviewer suggests.** We initially used 400 – 600 bp fragments to make the pilot library. **Our analysis of mutants recovered from the pilot library showed that inserts between 322 and 555 bp led to the successful recovery of mutants, and that there was no bias towards larger insert sizes in the mutants we recovered (Table 2).**

*4. How stable is the silencing phenotype? Does the level of silencing change over time? This would be an important consideration for how long strains can be maintained before being used for screening.*

**Response:** This question **has been addressed in the literature (Foda, et al., PMID: 26149683).** As we described in the introduction of this manuscript, the trigger silencing approach has been well-characterized in the literature, and it leads to long-term silencing of the gene of interest and the deposition of a repressive histone mark (Foda, et al., PMID: 26149683).

*5. Will gDNA fragments that hit promoters silence the associated gene?*

**Response:** Because the amount of intergenic sequence is relatively low (~50% of the genome is coding), we used gDNA fragments to make the plasmid RNAi library. This does mean that some library plasmids contain fragments of promoters, instead of coding sequences. Our analysis of the coding sequences of annotated genes showed that the library has genome wide coverage with respect to coding sequences. Notably, we did not recover any promoter fragments in our pilot screen. The distribution of endogenous small RNAs that are inversely correlated with gene expression is also consistent with coding sequences being most relevant to gene silencing, as endogenous small RNAs mapped to coding sequences (Zhang, et al., PMID: 19043551).

*6. Figures 6 and 7 lack scale bars.*

**Response:** We thank the reviewer for pointing this out. **In the revision, scale bars have been added to Figures 6 and 7.**

***Reviewer #3:*** *1) Author summary line 53: "enable forward genetics for the first time..."--as referenced by the authors, this is not quite true, since at least two prior "forward genetic" studies have been performed using overexpression libraries. Maybe better as "...the RNAi pathway to couple gene silencing with forward genetics for the first time..."*

**Response:** We agree and **we have changed the text in lines 54-55**, to state “to enable RNAi-based forward genetics for the first time.

*2) Introduction line 73: Despite the cited Cochrane Review, the statement "therapy is limited to nitroimidazoles" is incorrect--nitazoxanide is an effective alternative treatment for amebiasis. Maybe change to simply "therapy is limited".*

**Response:** We thank the reviewer for noticing this, and **we have corrected the statement in line 75,** “There is no vaccine and therapy is limited.”

*3) Results line 134 "a library of genes..." would be more accurate as "a library of gene fragments" or "genomic fragments".*

**Response:** We agree and **we have made this change in line 138,** to say, “a library of gene fragments.”

*4) Line 138: use of the word "mutants" here and in figure 1b is not accurate--these are not actually mutants. Although a little awkward, I think better as "targeted genes" or "silenced genes" throughout.*

**Response:** We thank the reviewer for pointing this out. **We changed the text in lines 142 - 143** to state “to compare the selected amoebae to control, non-selected amoebae, in order to identify gene fragments,” and **we changed the text in Figure 1b** to say “to quantitatively identify gene fragments.” Throughout the manuscript, in cases where we were referring to the plasmid library rather than transfected RNAi knockdown mutant cells, **we replaced the term “mutant” with the term “gene fragment,” for example in lines 377, 428 and 919.**

*5) Line 263 and throughout: I don't agree that growth rate is what's being measured, as the number of cells reflects both the rate of proliferation (i.e. growth rate) and persistence/longevity of cells in the culture. As the authors note, the increase in DAPI staining with the SG1 mutant indicates more average DNA content, which may indicate a higher rate of proliferation. That said, this conclusion could only be made with a more sophisticated experiment, such as using a vital dye (e.g. CFSE) and measuring the numbers/proportion of parasites that have divided 1, 2, 3x, etc. It would improve the accuracy to simply replace growth rate throughout with "cell number" or similar. If growth rate, then I think best to do a more sophisticated analysis. As above, I believe the importance of the paper is simply the method and library.*

**Response:** We acknowledge the reviewer’s point, and **we have removed the term “growth rate” throughout the manuscript.** We have also **added a new paragraph to the discussion, to explain the limitations of the assays we used, and how the results can be interpreted, in lines 342-348,** “In our analysis of slow growth mutants, we seeded cells at a set starting concentration, and then measured the number of cells at various time points thereafter. We defined slow growth phenotypes as those that resulted in significantly lower cell numbers over time, relative to control cells. In the case of cells overexpressing full-length SG1, an increased growth phenotype was also associated with a higher intensity of DAPI staining compared to control amoebae. The assays that we used do not formally distinguish between an altered rate of proliferation and altered longevity of individual cells in culture.”

*6) The methods indicate that t-tests were used to determine statistical significance. I believe that where multiple groups are being compared to the control the appropriate test would be an ANOVA or another test suitable for multiple comparisons.*

**Response:** We thank the reviewer for pointing out this issue, and we have since consulted with biostatisticians to determine a better way to analyze the multiple groups in our datasets. **In the revised manuscript, we used a two-way ANOVA with a post-hoc Dunnett’s multiple comparisons test to determine statistical significance. This is described in lines 646-648, and the data presented in Fig. 5a, 5c, 6d, S12a, S12c, S12e have been updated accordingly.** Note that it was not feasible to use ANOVA analysis for assessment of normalized data (shown in Fig. 5e, S12b, S12d, S12f); hence, ANOVA analysis was used for analysis of raw data and t-tests were used for analysis of normalized data.
